# Biomarkers Indicating Early Epithelial–Mesenchymal Transition Changes in Oral Epithelial Dysplasias: A Systematic Review

**DOI:** 10.3390/diagnostics16121891

**Published:** 2026-06-17

**Authors:** Diana-Ivette Rivera-Reza, Juan Carlos Cuevas-González, Alejandro Donohué-Cornejo, Alberto Rodríguez-Archilla, Luis Alberto Gaitán-Cepeda

**Affiliations:** 1 Department of Oral Medicine and Oral Pathology, Postgraduate Studies and Research Division, Faculty of Dentistry, National Autonomous University of Mexico, Mexico City 04510, Mexico; dianrvr@fo.odonto.unam.mx; 2 Instituto de Ciencias Biomédicas, Universidad Autónoma de Cd. Juárez, Av. Benjamín Franklin # 4960, Zona Pronaf, Ciudad Juárez 32315, Mexico; cuevas_gonzalez@hotmail.com (J.C.C.-G.); adonohue@uacj.mx (A.D.-C.); 3 Oral Medicine Unit, Department of Stomatology, Faculty of Dentistry, University of Granada, 18010 Granada, Spain; alberodr@ugr.es

**Keywords:** epithelial–mesenchymal transition, oral potentially malignant disorders, oral epithelial dysplasia, biomarker, E-cadherin, Vimentin

## Abstract

**Background/Objectives**. Oral epithelial dysplasia exhibits unpredictable behavior, prompting research to identify biomarkers that may help predict its progression to malignancy. This study aimed to ascertain the prognostic value of biomarkers indicative of the epithelial–mesenchymal transition (EMT) process using immunohistochemistry. **Methods**. A systematic review was conducted using PubMed data from 1978 to June 2026. Articles that employed immunohistochemistry to identify cells exhibiting epithelial–mesenchymal transition changes in oral epithelial dysplasia were included. Exclusion criteria included in vivo studies, book chapters, reviews, conference abstracts, and studies lacking population descriptions. The risk of bias was assessed using the “JBI Checklist for Critical Appraisal of Case Series”. **Results**. A total of 21 articles were included, analyzing 57 biomarkers: 34 epithelial, 19 mesenchymal, two cell-proliferation biomarkers, and two tumor-suppressor biomarkers. The sample sizes varied significantly between the studies. Most articles employed semiquantitative assessment, cell percentage, and immunostaining intensity, with 12 demonstrating low risk of bias. **Conclusions**. Studies with conclusive results and a low risk of bias suggest that E-cadherin and Vimentin are valuable biomarkers for identifying early EMT in OED. However, the lack of statistical support means that this assertion should be viewed with caution.

## 1. Introduction

Oral potentially malignant disorders (OPMDs) are specific morphological alterations of the oral epithelium. OPMDs are associated with an increased risk of oral cancer, particularly oral squamous cell carcinoma (OSCC) [1]. Clinically, OPMDs include leukoplakia, erythroplakia, oral submucosal fibrosis, and lichen planus [2,3]. Histopathologically, oral epithelial dysplasia (OED) carries the highest risk of malignant progression. OED is characterized by cytological changes, including cellular and nuclear pleomorphism, an increased nucleus-to-cytoplasm ratio, and aberrant mitosis. It also exhibits architectural changes, such as basal membrane duplication, loss of cellular cohesion, and premature keratinization [2]. Regarding classification systems, two approaches have been proposed for OED, based on qualitative or quantitative cytological or cytoarchitectural changes. The most widely used, the World Health Organization (WHO) classification, is based on the extent of dysplastic changes within the epithelial thickness: if the cytological and architectural changes are confined to the basal third, the grade is mild; extension to the middle third indicates moderate grade; and involvement of the entire epithelium signifies severe grade [3,4]. In contrast, the binary classifier classifies OED as low or high risk for malignant transformation based on the quantity and quality of cellular atypia [3,4].

The significance of OED, regardless of classification, lies in identifying patients at elevated risk for oral cancer. This enables vigilant surveillance, regular follow-up, and preventive intervention. Although severe/high-risk OED is associated with a greater risk of malignant transformation, OED remains unpredictable. Severe/high-risk OED may stay inactive throughout a patient’s life, while mild/low-risk OED can progress rapidly. Despite extensive attempts to identify highly specific and sensitive biomarkers for early malignancy detection, no reliable method or biomarker exists to identify OED patients at high risk of progression.

Recently, studies have focused on the biological mechanisms of OED that may precede malignant transformation. These studies highlight epithelial–mesenchymal transition (EMT), a fundamental and reversible process in carcinogenesis. In EMT, epithelial cells lose their characteristic phenotype and acquire a mesenchymal one. This shift is critical for invasion during the neoplastic epithelial process [5]. It was recently revealed that EMT confers properties to cancer stem cells, making them resistant to immunotherapy and chemotherapy [6]. EMT is also considered an initial step in the invasion–metastasis cascade [7]. Gradual cellular changes characterize EMT, with cells expressing a range of epithelial and mesenchymal markers. Before invading the stroma, epithelial cells are cuboidal and remain closely packed, showing positive E-cadherin and negative Vimentin expression. Hybrid-EMT cells take on a spindle shape and display weak cell-to-cell adhesion. These cells co-express E-cadherin and Vimentin. In the mesenchymal stage, neoplastic cells appear fibroblast-like, lose cell–cell junctions and E-cadherin expression, and exhibit increased Vimentin expression [8].

Hypothesizing that OEDs with a higher probability of malignant progression present early EMT-associated changes, the principal objective of the present study was to systematically review whether OEDs exhibit early EMT-related changes, which biomarkers are proposed, and, if so, their prognostic value. The identification of early EMT-related changes in OEDs can provide clinicians with a reliable tool to shorten the diagnostic process and improve treatment and follow-up decisions.

## 2. Materials and Methods

### 2.1. Study Type and Research Questions

A systematic review was conducted using MEDLINE/PubMed^®^, Cochrane, and Scopus from 1978 to June 2026. The review addressed which biomarkers most reliably identify the epithelial–mesenchymal transition (EMT) in oral epithelial dysplasia. The PICO strategy was as follows: Population = patients with oral epithelial dysplasia, defined by architectural and cytological changes linked to increased risk of oral squamous cell carcinoma; Intervention = assessment of EMT biomarkers; Comparison = no comparator due to study design; and Outcome = identification of EMT-related phenotypic changes in epithelial cells via biomarkers. The research design and conduct were carried out in accordance with the PRISMA guidelines by Page MJ, McKenzie JE, Bossuyt PM, et al. BMJ. 2021;372:n71. doi: 10.1136/bmj.n71 [9]. (Supplementary Materials).

### 2.2. Search Syntax

The MeSH terms and keywords were used to build a search syntax as follows: ((premalignant condition [Text Word]) OR (oral potentially malignant disorders [Text Word])) OR (premalignant lesions [Text Word])) OR (oral epithelial dysplasia [Text Word])) AND ((epithelial-mesenchymal transition [MeSH Terms]) OR (mesoderm [MeSH Terms])) OR (stroma [Text Word])).

### 2.3. Selection Criterion

Articles in English and Spanish that aimed to identify changes associated with the mesenchymal–epithelial transition in oral epithelial dysplasia were included in this review. In vivo studies, book chapters, review articles, conference or congress abstracts, studies in which the sample was not described, and studies in which biomarkers of epithelial–mesenchymal transition were not identified by immunohistochemistry were excluded or where the method of immunohistochemical interpretation was not specified.

### 2.4. Procedure

The articles identified using the search strategy were first reviewed to eliminate duplicates. The remaining articles were then reviewed by title and abstract to identify those that met the inclusion criteria for this review. The remaining articles were read in full. Two experts (LAGC/DIRR) independently reviewed the articles without knowledge of the other reviewer’s inclusion decisions. Disagreements were discussed until a consensus was reached. Articles were discarded if consensus was not achieved.

Available data were collected on sample size, the biomarkers used, the classification of OED (all measurement scales), the immunohistochemical assessment method (all measurement scales) and prognostic value. The data were compiled in a Microsoft^®^ Excel spreadsheet.

### 2.5. Risk of Bias

The risk of bias in the individual studies was assessed by two experts (LAGC/DIRR) using the “Checklist for Critical Appraisal of JBI Case Series” [10], which comprises 10 questions with the following options: yes, no, unclear, or not applicable. The risk of bias was classified based on the percentage of “Yes” responses: high when the percentage was less than 49%, moderate when 50–69%, and low when 70% or higher.

### 2.6. Effect Measures

Information reported in each included article was compiled; for immunohistochemical interpretation, this included findings reported as positivity, the location of the immunostaining, and the implications for prognostic value.

### 2.7. Synthesis Methods

The data was entered into a Microsoft^®^ Excel spreadsheet, and summary tables of the results were subsequently compiled without making any modifications or conversions to the measurements reported for each item included.

### 2.8. Reporting Bias Assessment and Certainty Assessment

The criteria established by the REMARK guidelines [11] were used to assess the studies included in the systematic review.

## 3. Results

**Figure 1:**
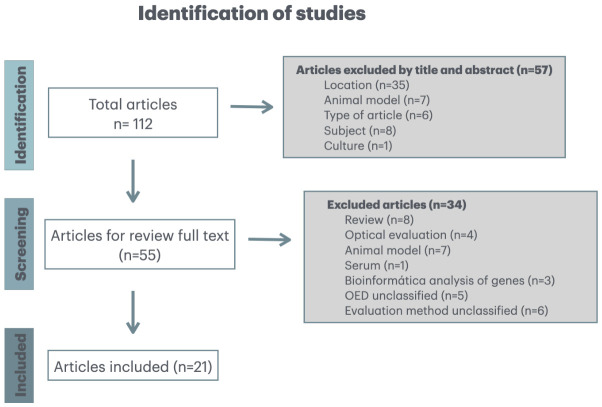
PRISMA CHARTFLOW.

The search process identified 112 studies. After reviewing the titles and abstracts, 57 articles were excluded for not meeting the selection criteria, including specific requirements such as the use of human samples and immunohistochemical analysis. The remaining 55 articles were reviewed in full. An additional 34 were excluded due to factors such as the use of animal models, lack of immunohistochemistry, or the presence of review articles. Thus, 21 primary research articles published between 1989 and 2026 [12,13,14,15,16,17,18,19,20,21,22,23,24,25,26,27] were included in the detailed analysis. The selection process and criteria are illustrated in Figure 1 and summarized in Table 1 and Table 2. Among the 21 selected articles, 785 cases were reported, with individual studies reporting sample sizes ranging from 7 [16] to 87 [22]. For OED classification, the methods varied: 11 articles used the WHO system (mild, moderate, and severe) [11,12,14,16,17,18,21,25,27,28,29,30]; four articles used a binary system (low risk; high risk) [20,21,27,28]; three did not specify OED grades and simply compared with OSCC [14,25,26]; one included only moderate dysplasia [16]; and one included only high-grade dysplasia [29].

In addition to the previously described article selection, 57 biomarkers were used, of which 34 were epithelial (Table 1), 19 were mesenchymal, two were cell-proliferation markers, and two were tumor-suppressor biomarkers (Table 2). Various methodologies were used to assess EMT immunoreactivity. In 12 articles, a semiquantitative approach was used to evaluate two aspects: immunoreaction intensity and the percentage of stained cells. The number of categories for the percentage of stained cells varied across reports: 0–3 [13,16,20,25,28,29,30,31,32], 0–4 [19], 0–5 [15], or 1–3 [26]. Four studies [12,17,22,23] provided descriptions (location and positivity). In one study, only the percentage of stained cells was reported [14], and a quantitative analysis was performed using the software [27]. Biomarkers used to predict progression to malignancy include CEA [12], EMA [12], EGF [12], E-cadherin [17,18,19], N-cadherin, Snail [19], Twist1 [18], Vimentin [17,20], MMP-9 [31], FGF-2 [27], FGFR-1 [27], ATG16L [13], podoplanin [20], and p53 [20].

Finally, the risk of bias was assessed in all 21 studies. A total of 12 (57.2%) were classified as having a low risk of bias [12,13,16,17,20,21,22,25,26,28,30,31], five (23.8%) with a moderate risk of bias [14,18,19,29,32], and in four (19.0%) studies, the risk of bias was high [15,23,24,27]. The detailed results are presented in Table 3.

## 4. Discussion

This systematic review examined the literature on EMT presence in OED using immunohistochemistry and assessed the utility of markers for identifying high-risk OED cases. EMT was first proposed by Greenberg and Hay in 1982 [33]. Understanding EMT and identifying related genes or products may indicate progression to malignancy, aiding early diagnosis and individualized strategies for OED and OSCC. Ha et al. proposed that most OED alterations occur in the shift from normal to precancerous state [34], when early EMT changes are significant. EMT is a complex, reversible process where epithelial tumor cells switch to a mesenchymal phenotype [14] and is linked to invasion, migration, and metastasis [17]. EMT involves events such as disruption of cell junctions, changes in cell shape, downregulation of epithelial markers, and increased expression of mesenchymal markers, which promote cell movement [18]. Repression of E-cadherin and induction of mesenchymal markers, such as N-cadherin and Vimentin, remain the gold standard for confirming EMT. Thus, identifying Vimentin presence and E-cadherin loss or reduction in OED is an effective approach to early EMT detection. This review identified promising biomarkers for the early stages of EMT development and progression.

E-cadherin is a key cell adhesion molecule that depends on calcium and is encoded by CDH1 gen. When E-cadherin is reduced, cancer cells may migrate more, grow faster, become more aggressive, invade tissues, spread, and have a poorer outcome. E-cadherin is also a strong prognostic marker. In low-grade OED, E-cadherin levels are similar to those in normal tissue, but they drop in OED and OSCC. Keeping the epithelial type is important. More loss and cytoplasmic redistribution of E-cadherin occur in severe or high-risk OED [17]. Also, when membrane E-cadherin is low, cytoplasmic N-cadherin is high in OED, suggesting EMT occurs in these cases [19]. Vimentin is a type III intermediate filament protein with a structural role in mesenchymal cells that participates in regulating migration, cell signaling, and cell attachment. Abnormal expression of this gene is associated with tumor invasion and metastasis, but it is not immunoreactive in normal buccal mucosa. Vimentin expression increases as OED progresses to OSCC [17]. Consequently, in OED, downregulation and translocation of E-cadherin, along with increased expression of Vimentin, occur during early epithelial–mesenchymal transition [17].

The present systematic review revealed that other biomarkers are also useful. Carcinoembryonic antigen (CEA) and epithelial membrane antigen (EMA) levels increase with the progression of dysplastic grade, whereas epidermal growth factor (EGF) levels decrease in high-risk OED [12]. Protein arginine methyltransferase 5 (PRMT5) is a highly promising biomarker. PRMT5 overexpression causes cellular hyperproliferation, whereas its knockdown is associated with reduced cell growth. PRMT5 is a type II arginine methyltransferase that regulates apoptosis, cell proliferation, and differentiation. PRMT5 overexpression is correlated with the loss of E-cadherin and cytokeratin 17 immunoexpression in OED, suggesting that cytoplasmic PRMT5 may play a role in early oncogenesis [14]. PRMT5 provides evidence that EMT may begin early in OED [13]. It is important to note that in normal epithelium, PRMT5 is weakly expressed, and there is no immunoexpression in basal layer cells [35]. TWIST1, a transcription factor, is a key regulator of epithelial–mesenchymal transition. Cytoplasmic immunoexpression of TWIST1 regulates proliferation, migration, and invasion and serves as a prognostic marker for oral tongue squamous cell carcinoma. TWIST1 is expressed in OED [18]. SNAIL1, another transcription factor, correlates with the loss of E-cadherin from the cell membrane in OED, indicating EMT progression. Downregulating or silencing SNAIL1 plays a key role in suppressing EMT and in inhibiting cell migration. It also affects cell proliferation and is linked to clinical prognostic factors, including aggressiveness, metastasis, and poor survival. SNAIL1 may be a promising therapeutic target for preventing tumor progression [19]. Fibroblast growth factor (FGF)-2 and fibroblast growth factor receptor (FGFR)-1 are associated with tumor invasiveness, cell proliferation, angiogenesis, and metastasis and have been linked to high-grade OEDs. Increased expression of FGF-2 and FGFR-1 correlates with high-grade OEDs [27]. A key event in EMT is the loss of E-cadherin through direct repression of the CDH1 gene by transcription factors, such as ZEB1/ZEB2 [36,37]. The zinc-finger E-box-binding homeobox (ZEB) family in humans, including ZEB1 and ZEB2, are master regulators of EMT. High ZEB1 and ZEB2 expression leads to reduced transcription of epithelial markers and increased expression of the mesenchymal markers Vimentin and N-cadherin [36,37]. ZEB1 suppresses cell polarity factors and basement membrane synthesis and activates the expression of matrix metalloproteases 1, MMP-9, and MMP- 14, promoting basement membrane remodeling and invasion into surrounding tissues [38]. These transcription factors have been implicated in EMT in various cancers; however, their potential roles in OPMDs, specifically OED, remain unexplored.

Various processes occurring within subepithelial tissues may facilitate EMT, even if they do not directly induce it. These processes are considered significant because they have the potential to support EMT. A notable example is angiogenesis, which contributes to tumor growth. Substantial evidence indicates that angiogenesis precedes the full development of cancer. Certain inflammatory cells secrete matrix metalloproteinase-9 (MMP-9), vascular endothelial growth factor (VEGF), and cyclooxygenase-2 (COX-2), which promote angiogenesis. The direct involvement of matrix metalloproteinases (MMPs) in the initiation and progression of epithelial cancers is well-documented. Elevated protease levels facilitate the degradation of the extracellular matrix, enabling tumor cells to migrate and disseminate via the circulatory and lymphatic systems. MMP-9 initiates angiogenesis by activating factors such as VEGF and fibroblast growth factor (FGF). While MMP-9 expression is low in the basal layer of normal epithelia, it increases significantly as OED progresses. The presence of MMP-9 in dysplastic lesions may indicate its role in early alterations during the malignant transformation of the oral epithelium [31]. The potential involvement of myofibroblasts in the progression of OED to malignancy has been previously investigated. Although findings were inconclusive, immunohistochemical analysis revealed that alpha-smooth muscle actin (α-SMA) was expressed, indicating the presence of myofibroblasts. Myofibroblasts derived from tumor-associated connective tissue are more effective in promoting tumor development than normal fibroblasts from healthy tissues. It has been reported that high-risk OED exhibits significantly higher α-SMA expression than low-risk epithelial dysplasia (LRED) in oral submucous fibrosis [32].

Our results showed that the biomarkers studied included cadherins, TWIST, Vimentin, and cytokeratin 17, consistent with previous reviews [39,40], as well as p53 and Ki67 [40]. Although immunohistochemistry (IHC) is recognized as a reliable and standardized molecular biology technique, the lack of consensus on the parameters used to evaluate it [41,42,43] significantly affects the repeatability and validity of studies. Our results are consistent, indicating a highly heterogeneous, broad system for evaluating immunohistochemical expression. Semiquantitative evaluation techniques have been proposed to measure two factors: the intensity of immunoreaction and the quantity of immunolabeled cells. These methods include the H-score system [13], the Kellermann system [26,28,29], the Allred scores [15], and the Tuxhorn method [25,32]. A third factor, cell-specific localization of each biomarker, has also been proposed [17,22,23]. These facts highlight the essential need to establish a standardized evaluation method for research projects, enabling more efficient comparisons of results and, ultimately, the development of quantifiable scales for clinical use. The lack of a standardized method for assessing immunoreactivity limits the comparability of the results across studies. This methodological heterogeneity reflects the lack of an international consensus on a validated standard. Recently, The Scientific and Regulatory Policy Committee of the Society of Toxicologic Pathology [44] posited that a “visual scoring system for IHC assays should be definable, reproducible, and produce meaningful results,” and predicted that this would improve the robustness and reproducibility of chromogenic IHC studies. Until such standardization is achieved, methodological diversity will continue to hinder robust statistical analyses. Immunoreactivity assessment systems can be divided into two types: semiquantitative methods, such as visual scoring (nominal or binary, ordinal scoring, percent positive, H-score, and Allred score), and quantitative analysis, which uses automated digital analysis [44]. Recent developments in computational software have facilitated the application of quantitative metrics for assessing immune responses. The precision of area estimations could be enhanced through automated IHC analysis. Approaches based on artificial intelligence might assist in overcoming this issue.

If we consider the risk of bias as an indicator of the quality of the results obtained, we can conclude that CEA, EMA [12], PRMT-5 [14], E-cadherin [14,20], Vimentin [17,20,25], podoplanin [20], p53 [20], Ki67 [20] SMA [28] and MMP-9 [31] are the most reliable biomarkers for identifying early changes in EMT in OED and suggestive of the onset of progression to malignancy. Although numerous biomarkers have been described and studied, a risk-of-bias analysis can identify those with the strongest evidence for use as indicators of OED progression. Studies with low risk of bias reported that the immunohistochemical expression of CEA, EMA, and MMP-9 increased in severe/high-risk OED [12,31], whereas the immunohistochemical expression of EGF and E-cadherin decreased [12,16] (Table 4). In contrast, data on biomarkers for myofibroblast identification are inconclusive [25,26,28,29].

The present study had some limitations. Most of the articles reviewed and analyzed were cross-sectional studies, lacking follow-up and thus unable to establish causality. Few studies have been conducted in hospitals [14,32] or in cohort studies [16], and clinical parameters related to disease progression, such as location, TNM stage, follow-up time, and treatment, have been incorporated [30]. These variables are important for the study. The most crucial factor is the presence of OSCC, which can confirm disease progression. It is essential to consider the design of longitudinal studies, as this will enable statistical confirmation of EMT’s utility as a prognostic marker. However, the lack of standardization in immunoreactivity assessment systems is a weakness. Because values vary by method, from qualitative to quantitative assessments or combinations of both, it is not possible to perform a statistical test. An alternative is to pool raw data from all included articles and analyze them as a whole. A systematic review with meta-analysis was published to identify the early molecular mechanisms associated with or contributing to the malignant transformation of potentially precancerous oral lesions, specifically leukoplakia [45]. The authors identified longitudinal articles to avoid cross-sectional limitations. Statistical analysis used a pooled proportion design, grouping biomarkers into the 10 hallmarks of cancer. Their results suggest that immunohistochemical biomarkers of cell proliferation, EMT, and molecular mechanisms of immune evasion are strongly associated with progression to malignancy, as supported by longitudinal studies [45]. For EMT, which is important for the present study, findings show that epithelial cells’ ability to activate invasion and metastasis mechanisms increases the likelihood of malignancy in oral leukoplakia by 3.4 times. It identified E-cadherin, podoplanin, *β*-catenin, and Vimentin as EMT biomarkers with prognostic value [45]. This report supports our study, in which, despite the methodological limitations of some studies, we reached the same conclusion. However, a limitation of the study by González-Ruiz I et al. [45] is that, because it used pooled prevalence analysis, it was not possible to identify a specific biomarker. However, our systematic review did not identify such biomarkers.

## 5. Conclusions

Immunohistochemistry remains a useful and reliable tool. Studies with conclusive results and a low risk of bias suggest that E-cadherin and Vimentin are the most valuable biomarkers for identifying early EMT in OED. However, the lack of statistical support means that this assertion should be viewed with caution. If possible, the immunohistochemical panel could be expanded to include CEA, EMA, EGF, podoplanin, p53, and Ki67; with greater reservation, finally, myofibroblasts are not useful in OEDs, similar to CK19, MTAP, and EMMP.

The primary EMT processes include loss of cohesion and increased motility. Results of the systematic review confirm that the most promising biomarkers are E-cadherin, an adhesion molecule, and Vimentin, a cytoskeleton component linked to motility. Expression patterns of E-cadherin, N-cadherin, and SNAIL1 suggest EMT is present in OED. This indicates EMT plays a critical role in the development and progression of oral cancer. Research protocols should be developed to confirm this hypothesis. Long-term follow-up is needed to assess prognosis and predict the malignant potential of premalignant lesions. Supported by the concept of personalized precision medicine, which is based on identifying markers that can be utilized in the clinical setting for early diagnosis, prognosis, and targeted therapy [36], EMT has been proposed as a therapeutic approach because several molecules, including transcription factors, have been identified as key regulators of the EMT process [36]. However, no EMT biomarkers have been clinically tested as treatment targets; therefore, their utility as therapeutic targets in OED should be viewed with caution. Future clinical studies should be designed to evaluate the possible usefulness of molecular factors in the early treatment of high-risk OED.

## Figures and Tables

**Table 1:** Characteristics of the epithelial biomarkers related to EMT in OED.

Author (Year)	OED Classification (*n*)	Biomarkers	Evaluation	Results
Sano K, 1989 [12]	WHO (66; 18 mild, 32 moderate, 16 severe)	CEA EMA EGF	Positive immunoreactivity indexes.	CEA and EMA immunoexpressions increase with severity grade. EGF decreases with severity grade.
Nomura H et al. [13] (2009)	WHO (31; 5 mild, 19 moderate, 7 severe)	ATG16L	Semiquantitative H-score system. Intensity (0–3) and percentage of stained cells.	Heterogeneous expression. High cytoplasmic expression in mild OED. Cytoplasmic membrane overexpression in moderate/severe OED.
Amano Y et al. [14] (2018)	WHO (8)	PRMT5 CK13 CK17 E-cadherin	Quantitative. Percentage of stained cells: negative, intermediate, and positive.	PRMT5: Positive immunoreactivity. CK13: Positive immunoreactivity. CK17: Positive immunoreactivity. E-cadherin: Decreased immunoreaction. Vimentin: Increased immunoreaction. PRMT5: Cytoplasmic expression in the early phase of oncogenesis; nuclear expression in the late phase.
Rajeswari P et al. [15] (2021)	WHO (30; 10 mild, 10 moderate, 10 severe)	CK19	Allred score. Percentage of stained cells [0–5] + intensity [0–3]).	Inconclusive.
Amano Y et al. [16] (2022)	WHO (7; moderate)	MTAP PRMT 1, 5 p16 E-cadherin Slug	Semiquantitative. Percentage of cells (0–3) + intensity (0–3).	Inconclusive.
Puneeta N et al. [17] (2022)	WHO (60; 20 mild, 20 moderate, 20 severe)	E-cadherin	Qualitative–quantitative. Percentage of stained cells plus extent, location, and intensity.	Loss of E-cadherin expression is related to severe OED.
de Morais EF et al. [18] (2022)	WHO (47; 13 mild-moderate, 34 severe)	E-cadherin N-cadherin Twist1	Semiquantitative.	Twist: nuclear and cytoplasmic expression, basal and parabasal. E-cadherin: decreased immunoexpression. N-cadherin: elevated immunoexpression.
de Morais EF et al. [19] (2023)	WHO (47; 13 mild-moderate, 34 severe)	Snail1 E-cadherin N-cadherin	Semiquantitative. Percentage of stained cells (0–4) + intensity (0–3).	Snail: High and progressive expression levels. E-cadherin: Loss of membrane expression.
Miguel AFP et al. [20] (2023)	Binary (61; 37 low risk, 23 high risk)	Podoplanin, E-cadherin	Percentage of stained cells and pattern.	Loss of E-cadherin expression, intense or continuous expression of podoplanin.
Tokozlu B et al. [21] (2024)	Binary (23; 14 low risk, 9 high risk)	Snail, E-cadherin, CD133, CD44	Semiquantitative H-score system. Intensity (0–3) and percentage of stained cells.	Loss of E-cadherin expression; no biomarker correlated with the degree of OED.
Yim IS et al. [22]	WHO (87 mild & moderate; 29 progression)	E-cadherin, *β*-catenin	Qualitative location, and intensity (0–3), high or low expression.	Inconclusive.
Condurache Hrițcu OM et al. [23] 2025	OPMDs (49)	Maspin, *β*-catenin	Qualitative, location (nuclear, cytoplasmic, and membrane expression), intensity (0–3).	Maspin is strongly expressed in the cytoplasm and nucleus; *β*-catenin is strongly expressed in the membrane and cytoplasm. Prospects for risk stratification.
Ghose S et al. [24] 2025	WHO (25) Non-specified	PAR3, SCRIBBLE, DL67	Semiquantitative, intensity (0–3), Intensity Scoring Unit (ISU), ratio of observed weight to the maximum possible score.	PAR3 absent, SCRIBBLE 70% negative, DL67 70% lost. Propose an objective classification of OEDs and risk stratification in OMPDs.

*n* = sample, CEA = carcinoembryonic antigen, EMA = epithelial membrane antigen, EGF = epidermal growth factor, OED = oral epithelial dysplasia, WHO = World Health Organization, ATG16L = autophagy-related 16-like 1, PRMT-5 = protein arginine methyltransferases-5, CK = cytokeratine, MTAP = methylthioadenosine phosphorylase, OPMD = oral potentially malignant disorders.

**Table 2:** Characteristics of the mesenchymal biomarkers related to EMT in OED.

**Author** **(Year)**	**OED** **Classification** **(*n*)**	Biomarkers	**Evaluation Method**	**Results**
Etemad-Moghadam S et al. [25] (2009)	WHO (15)	α-SMA, Desmin, Vimentin	Tuxhorn method: percentage of stained cells (0–3) and intensity (0–3) = index (negative, low, high).	No myofibroblasts were identified.
Seifi S et al. [26] (2010)	WHO (18; 6 mild, 6 moderate, 6 severe)	α-SMA	Kellerman et al. method: % of stained cells (1–3) and staining pattern (focal, network, spindle).	Inconclusive.
Amano Y et al. [14] (2018)	WHO (8)	Vimentin	Quantitative % stained cells. <5% = negative; >5% = positive.	Increased expression of Vimentin.
Mariz BALA et al. [27] (2018)	Binary (36)	FGF 2, FGFR 1	Quantitative Aperio positive pixel. Tumor score = (weak percentage × 1) + (moderate percentage × 2) + (strong percentage × 3).	Immunoexpression was positive in basal and suprabasal epithelial layers in low-risk OED, while in high risk it was more intense and covered the upper layers. Useful to predict prognosis.
Arora M et al. [30] (2018)	WHO (60; 20 mild, 20 moderate, 20 severe)	EMMPRIN (CD 147)	Quantitative. Intensity (0–3) and area.	Inconclusive.
Bhattacharjee K et al. [29] (2018)	WHO (20; severe)	α-SMA	Kellermann et al. method. Percentage of cells (0–3). and staining patterns.	Heterogeneous expression.
Deepa J et al. [31] (2018)	WHO (30; 10 mild, 10 moderate, 10 severe)	MMP-9	Quantitative. Q-Winstandard program. Intensity (0–3) and % of stained cells (0–3).	Expression of MMP-9 and vascularity increase, suggest the effect of MMP-9 on angiogenesis in tumorigenesis.
Gadbail AR et al. [28] (2018)	Binary (50; 28 low risk, 22 high risk)	Ki67, CD105, α-SMA Binary	Modified Tuxhorn et al. method. % of stained cells (0–3).	Ki-67 and α-SMA show increased expression in high-risk OED.
Pinisetti S et al. [32] (2022)	WHO (15: 5 mild, 5 moderate, 5 severe)	α-SMA	Kellermann et al. method. % of cells (0–3) and staining patterns.	Inconclusive.
Amano Y et al. [16] (2022)	WHO (7: moderate)	Vimentin, Laminin5	Semiquantitative, % of cells (0–3) + intensity (0–3).	Inconclusive.
Puneeta N et al. [17] (2022)	WHO (60: 20 mild, 20 moderate, 20 severe)	Vimentin	Quantitative. Intensity, location, % of stained cells and extent.	Vimentin expression increases in severe OED.
Miguel AFP et al. [20] (2023)	Binary (61; 37 low risk, 23 high risk)	Vimentin, Ki67, p53, OTPN, IL-6	Percentage of stained cells and patterns.	High immunoexpression of Vimentin; high Ki67 expression in high-risk OED; high p53 expression in OED with intense IL-6 expression; high OTPN expression in high-risk OED.
Condurache Hrițcu OM et al. [23] 2025	OPMDs (49)	MMP-14	Qualitative. Location (nuclear, cytoplasmic, and membrane expression), and intensity (0–4).	Low cytoplasmic expression of MMP-14, suggesting a reduced level of extracellular matrix remodeling.

*n* = sample, OED = oral epithelial dysplasia, WHO = World Health Organization, MMP = matrix metalloproteinases, FGF = fibroblast growth factor, FGFR = fibroblast growth factor receptor, EMMPRIN = extracellular matrix metalloproteinase inducer, OTPN = glycol-phosphoprotein osteopontin.

**Table 3:** Risk-of-bias analysis.

	Article	Q1	Q2	Q3	Q4	Q5	Q6	Q7	Q8	Q9	Q10	% of Yes	Risk
Epithelial biomarkers	Sano K [12]	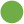	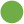	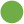	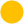	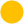	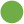	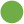	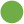	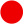	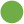	70	LOW
	Nomura H et al. [13]	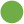	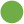	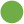	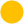	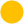	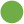	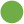	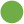	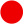	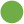	70	LOW
	Amano Y et al. [14]	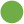	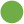	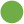	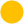	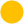	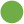	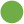	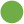	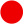	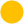	60	MODERATE
	Rajeswari P et al. [15]	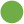	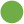	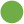	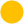	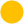	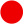	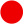	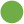	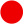	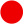	40	HIGH
	Amano Y et al. [16]	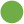	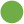	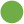	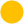	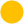	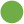	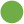	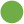	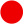	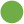	70	LOW
	Puneeta N et al. [17]	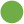	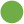	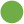	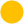	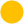	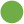	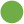	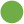	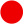	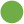	70	LOW
	de Morais EF et al. [18]	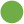	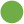	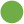	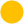	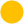	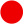	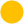	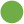	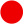	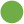	50	MODERATE
	de Morais EF et al. [19]	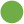	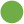	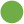	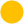	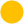	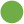	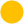	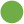	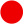	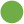	60	MODERATE
	Miguel AFP et al. [20]	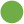	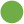	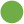	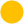	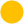	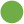	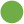	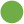	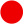	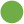	70	LOW
	Tokozlu B et al. [21]	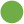	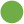	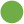	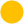	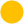	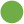	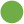	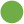	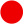	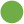	70	LOW
	Yim IS et al. [22]	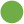	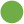	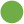	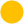	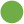	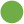	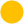	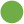	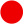	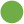	70	LOW
	Condurache Hrițcu OM et al. [23]	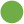	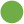	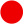	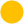	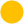	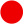	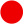	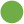	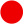	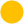	30	HIGH
	Ghose S et al. [24]	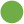	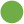	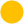	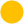	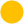	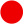	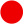	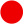	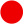	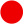	20	HIGH
Mesenchymal biomarkers	Etermad-Moghadam S et al. [25]	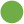	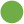	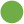	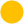	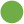	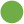	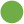	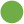	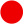	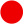	70	LOW
	Seifi S et al. [26]	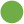	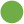	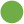	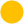	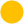	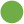	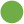	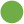	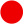	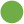	70	LOW
	Mariz BALA et al. [27]	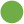	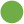	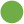	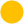	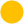	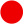	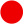	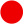	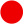	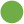	40	HIGH
	Arora M et al. [30]	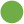	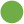	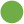	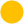	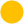	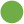	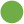	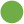	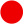	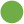	70	LOW
	Bhattacharjee K et al. [29]	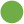	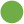	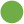	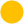	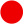	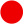	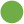	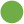	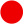	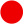	50	MODERATE
	Deepa J et al. [31]	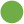	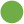	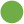	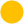	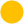	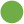	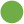	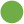	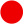	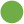	70	LOW
	Gadbail AR et al. [28]	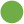	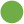	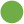	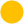	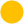	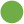	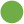	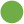	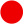	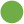	70	LOW
	Pinisetti S et al. [32]	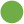	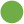	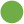	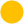	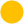	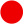	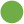	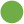	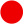	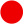	50	MODERATE

Q1. Were there clear criteria for inclusion in the case series? Q2: Was the condition measured in a standard, reliable way for all participants included in the case series? Q3: Were valid methods used for identification of the condition for all participants included in the case series? Q4: Did the case series have consecutive inclusion of participants? Q5: Did the case series have complete inclusion of participants? Q6: Was there clear reporting of the demographics of the participants in the study? Q7: Was there clear reporting of clinical information of the participants? Q8: Were the outcomes or follow up results of cases clearly reported? Q9: Was there clear reporting of the presenting site(s)/clinic(s) demographic information? Q10: Was statistical analysis appropriate? U: unclear (yellow), Y: yes (green), and N: no (red).

**Table 4:** Useful biomarkers to identify EMT in OED according to bias risk.

LOW RISK OF BIAS	
CEA, EMA, EGF [12]	The expression of CEA and EMA increases in severe/high-risk OED, while that of EGF decreases. Useful for confirming the severity of OEDs.
ATG16L [13]	Heterogeneous expression. High cytoplasmic expression in mild cases and overexpression in the plasma membrane in moderate/severe OED.
E-cadherin [17,20,21]	The loss of E-cadherin expression is associated with OED progression.
MMP-9 [31]	MMP-9 expression shows a progressive increase, suggesting an effect on angiogenesis and a possible role in tumorigenesis.
Ki67, a-SMA [32]	Ki67 and a-SMA expression progressively increase with OED grade.
Ki67, IL-6, Vimentin, p53, OTPN, podoplanin [20]	Elevated expression of Vimentin; Ki67 was significantly higher in high-risk cases; p53 expression was significantly higher in cases with intense IL-6 expression; the percentage of Ki67 nuclear positivity correlated with p53; and OTPN expression was higher in high-risk cases. Intense or continuous expression of podoplanin.

## Data Availability

The data presented in this study are available upon request from the corresponding author.

## Supplementary Materials

The following supporting information can be downloaded at https://www.mdpi.com/article/10.3390/diagnostics16121891/s1, Supplementary Materials: PRISMA 2020 Checklist.

## Abbreviations

The following abbreviations are used in this manuscript:

OPMDs	Oral potentially malignant disorders
OED	Oral epithelial dysplasia
OSCC	Oral squamous cell carcinoma
EMT	Epithelial–mesenchymal transition
CEA	Carcinoembryonic antigen
EMA	Epithelial membrane antigen
EGF	Epidermal growth factor
WHO	World Health Organization
ATG16L	Autophagy-related 16-like 1
AID	Activation-induced cytidine deaminase
hTERT	Human telomerase reverse transcriptase
PRMT-5	Protein arginine methyltransferases-5
CK	Cytokeratine
MTAP	Methylthioadenosine phosphorylase
MMP	Matrix metalloproteinases
TIMP	Tissue inhibitor of metalloproteinases
FGF	Fibroblast growth factor
FGFR	Fibroblast growth factor receptor
EMMPRIN	Extracellular matrix metalloproteinase inducer
OTPN	Glycol-phosphoprotein osteopontin
ISU	Intensity Scoring Unit
